# Bloodletting has no effect on the blood pressure abnormalities of hyperandrogenic women taking oral contraceptives in a randomized clinical trial

**DOI:** 10.1038/s41598-021-01606-7

**Published:** 2021-11-11

**Authors:** Manuel Luque-Ramírez, Andrés E. Ortiz-Flores, Lía Nattero-Chávez, M.Ángeles Martínez-García, María Insenser, Francisco Álvarez-Blasco, Elena Fernández-Durán, Alejandra Quintero-Tobar, Sara de Lope Quiñones, Héctor F. Escobar-Morreale

**Affiliations:** 1 Diabetes, Obesity, and Human Reproduction Research Group, Instituto Ramón y Cajal de Investigación Sanitaria (IRYCIS) and Centro de Investigación Biomédica en Red de Diabetes y Enfermedades Metabólicas Asociadas (CIBERDEM), Instituto de Salud Carlos III, Madrid, Spain; 2 University of Alcalá, Alcalá de Henares, Madrid, Spain; 3 Department of Endocrinology and Nutrition, Hospital Universitario Ramón y Cajal, Carretera de Colmenar Viejo, km 9.1, 28034 Madrid, Spain; 4 Department of Endocrinology and Nutrition. Hospital, Universitario de Torrejón, Torrejón de Ardoz, Madrid, Spain

**Keywords:** Endocrinology, Cardiovascular diseases, Endocrine system and metabolic diseases, Metabolic disorders, Reproductive disorders

## Abstract

Normoferritinemic women with functional hyperandrogenism show a mild iron overload. Iron excess, hyperandrogenism, and cardioautonomic dysfunction contribute to blood pressure (BP) abnormalities in these patients. Furthermore, combined oral contraceptives (COC) prescribed for hyperandrogenic symptoms may worse BP recordings. Iron depletion by phlebotomy appears to lower BP in other acquired iron overload conditions. We aimed to determine the effect of iron depletion on the office BP, ambulatory BP monitoring, and frequency of hypertension in patients with functional hyperandrogenism submitted to standard therapy with COC. We conducted a phase 2 randomized, controlled, parallel, open-label clinical trial (NCT02460445) in adult women with functional hyperandrogenism including hyperandrogenic polycystic ovary syndrome and idiopathic hyperandrogenism. After a 3-month run-in period of treatment with 35 µg ethinylestradiol plus 2 mg cyproterone acetate, participants were randomized (1:1) to three scheduled bloodlettings or observation for another 9 months. Main outcome measures were the changes in office BP, 24-h-ambulatory BP, and frequency of hypertension in both study arms. From June 2015 to June 2019, 33 women were included in the intention-to-treat analyses. We observed an increase in mean office systolic BP [mean of the differences (MD): 2.5 (0.3–4.8) mmHg] and night-time ambulatory systolic BP [MD 4.1 (1.4–6.8) mmHg] after 3 months on COC. The percentage of nocturnal BP non-dippers also increased, from 28.1 to 92.3% (*P* < 0.001). Office and ambulatory BP did not change throughout the experimental period of the trial, both when considering all women as a whole or as a function of the study arm. The frequency of the non-dipping pattern in BP decreased during the experimental period [OR 0.694 (0.577–0.835), *P* < 0.001], regardless of the study arm. Decreasing iron stores by scheduled bloodletting does not override the BP abnormalities caused by COC in women with functional hyperandrogenism.

## Introduction

Observational studies report a higher rate of hypertension in premenopausal patients with functional hyperandrogenism ‒ such as those with polycystic ovary syndrome (PCOS)—than in non-hyperandrogenic women^1^. The presence of weight excess, sympathetic hyperactivity, insulin resistance, and hyperandrogenemia by itself, are among the main contributors to the high prevalence of arterial hypertension in these otherwise young women^2–4^. Arterial hypertension in women with PCOS is a very relevant issue. A recent meta-analysis including cohort and case–control studies in over 30,000 and 130,000 women with or without PCOS, respectively, shows a statistically significant greater rate of cerebrovascular events in the former compared to their non-hyperandrogenic counterparts^1^. But even more concerning, the mainstay of treatment for hyperandrogenic symptoms — namely combined oral contraceptives (COC) — can exert a deleterious effect on blood pressure (BP) regulation both in the general population^5^ and in women with functional hyperandrogenism^6,7^. In keeping with this fact, international guidelines do not recommend the routine use of COC in women with hypertension, even if it is adequately controlled^8^.

Women with functional hyperandrogenism may show a mild iron overload, as defined by increased ferritin levels within normal range, compared with age- and body mass-matched non-hyperandrogenic women^9^. This fact is related to reduced circulating hepcidin levels — the key hepatic regulator of intestinal iron absorption — as a direct consequence of insulin resistance and androgen excess^9,10^. Iron excess could also contribute to hypertension. Tissue iron excess disrupts redox homeostasis leading to oxidative stress^11^. Reactive oxygen species facilitate endothelial dysfunction, aldosterone and mineralocorticoid actions, and inflammation, contributing to arterial hypertension^12^. In conceptual agreement, iron depletion by phlebotomy appears to lower BP in patients with the metabolic syndrome^13^. However, the long-term effects of bloodletting on BP or cardiovascular risk are somehow controversial beyond the amelioration of hyperviscosity states^14,15^. Furthermore, a short-term randomized clinical trial (RCT) comparing COCs with a single phlebotomy during 3 months of follow-up did not find any significant effect of bloodletting on the BP of women with PCOS^16^.

To provide new insights on this important issue for clinical practice, we here report the findings on office and ambulatory BP recordings of a RCT aiming to study the effects of decreasing iron tissue depots by scheduled bloodletting, in women with functional hyperandrogenism submitted to standard treatment with COCs. The main objectives of the present preplanned study were to determine the effect of iron depletion on the office BP, ambulatory BP monitoring, and frequency of hypertension in patients with functional hyperandrogenism submitted to standard therapy with COCs. As an exploratory aim, we also analyzed the impact of the scheduled bloodletting program on the cardiovascular autonomic function of these women.

## Results

**Figure 1:**
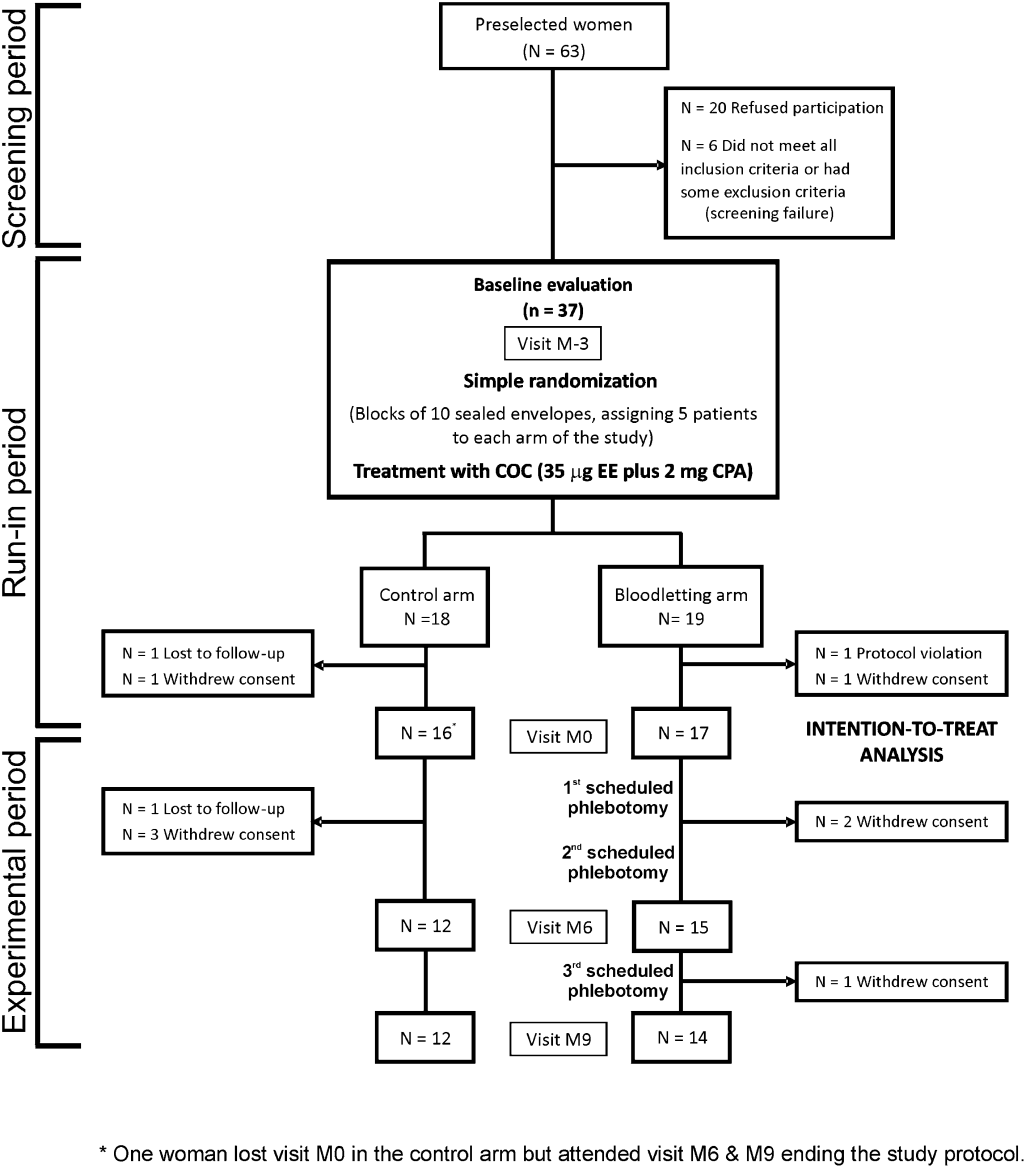
Flow chart of the study.

Figure 1 shows the flow chart of the trial. From June 2015 to June 2019, we screened 63 consecutive women, although 26 of them did not participate in the study because of different reasons. The remaining 37 women were randomized, but four of them did not complete the run-in period and were excluded from later analyses. Finally, 33 women were entered the trial, with 26 of them completing the study. Twenty-one (64%) patients had hyperandrogenic PCOS, whereas 12 (36%) had idiopathic hyperandrogenism^17^.

### ***Baseline characteristics (month ***− ***3 visit)***

**Figure 2:**
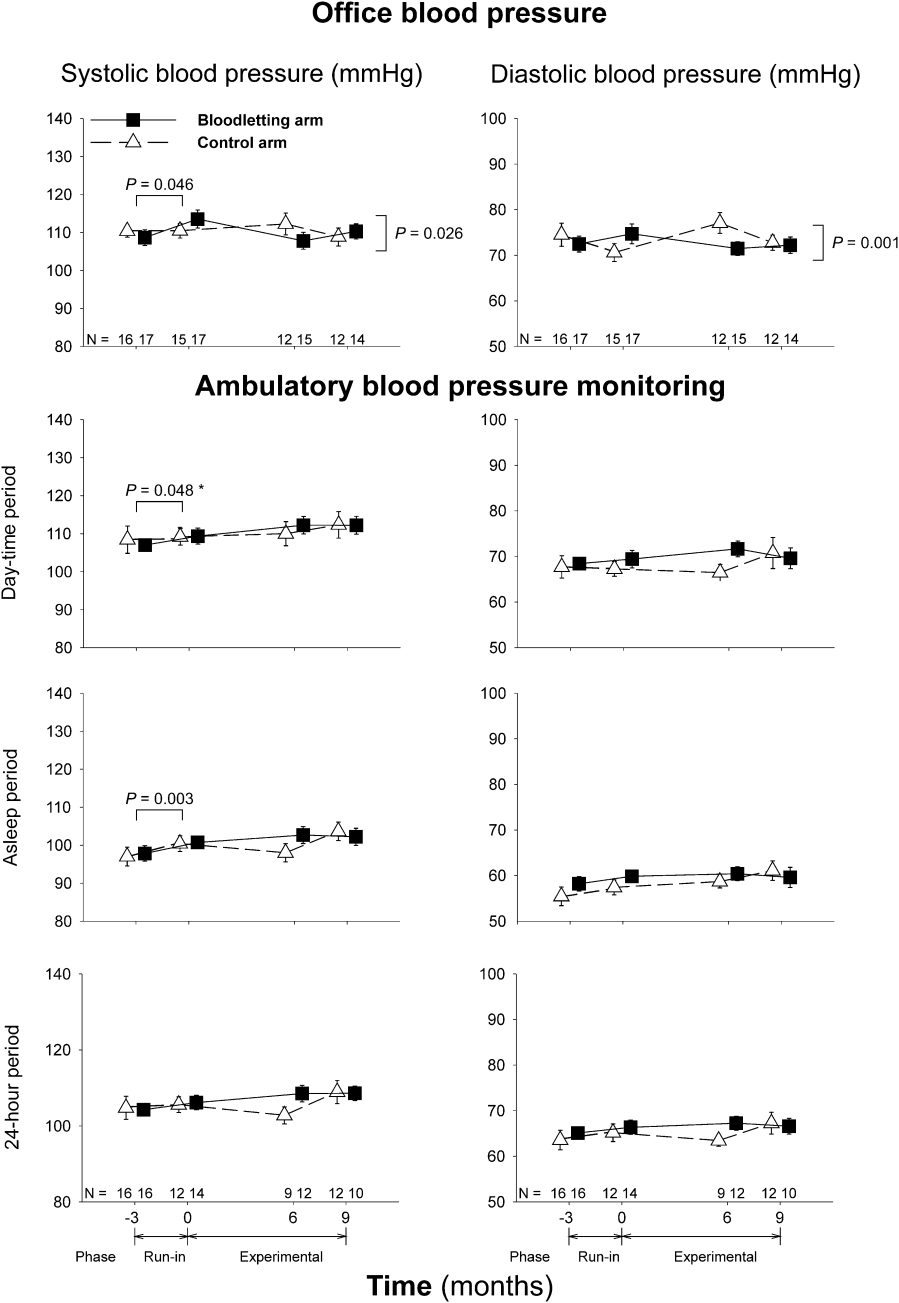
Changes in office blood pressure and ambulatory blood pressure monitoring recordings throughout the trial. Data are shown as means (SEM) of the patients remaining at each visit of the trial (figures above the x-axis) even though we conducted intention-to-treat statistical analyses. *Significant changes observed during the run-in phase in the whole group of participants after introducing the presence of obesity as between-subjects covariate. To analyze changes during the experimental phase, data were submitted to a repeated-measures general linear model.

The baseline characteristics of the women allocated to the experimental and observation arms of treatment were similar (Table 1). There were no differences between arms in those patients who completed all the study visits either (Table 1). At baseline, data were available for all office BP recordings, Valsalva tests and 30:15 ratios, and all but one (97.0%) ABPM recordings and E/I ratios. Mean BP and cardioautonomic function tests values are shown in Fig. 2 and Table 2, respectively. Two different women presented with office hypertension and hypertension while asleep according to ambulatory BP levels (Table 3). Nine women (28.1%) showed a non-dipping pattern in their nocturnal BP (Table 3). No women had resting tachycardia or orthostatic hypotension at baseline.

**Table 1:** Baseline characteristics of the hyperandrogenic patients in the experimental and control arms of the trial, both by intention-to-treat analysis and restricted to those patients who completed the trial.

	Intention-to-treat		*P*	Women completing the trial		*P *
	Experimental arm	Control arm		Experimental arm	Control arm	
	(n = 17)	(n = 16)		(n = 14)	(n = 12)	
Age (years)	25 ± 7	25 ± 6	0.813	26 ± 7	25 ± 6	0.779
	(21 to 29)	(22 to 28)		(22 to 30)	(21 to 29)	
Body mass index (kg/m^2^)	29.6 ± 8.1	28.3 ± 8.1	0.636	28.5 ± 7.0	28.8 ± 6.1	0.893
	(25.4 to 33.8)	(24.0 to 32.6)		(24.5 to 32.5)	(24.9 to 32.7)	
Waist circumference (cm)	90 ± 16	87 ± 20	0.634	90 ± 17	88 ± 17	0.804
	(82 to 98)	(76 to 98)		(80 to 100)	(77 to 99)	
Frequency of obesity, n (%)	8 (47)	6 (38)	0.579	6 (43)	5 (42)	0.951
	(26 to 69)	(19 to 61)		(21 to 67)	(19 to 68)	
Total testosterone (nmol/L)	2.6 ± 1.1	2.9 ± 1.0	0.459	2.6 ± 1.1	2.9 ± 1.0	0.552
	(2.1 to 3.2)	(2.3 to 3.4)		(2.0 to 3.2)	(2.2 to 3.5)	
Calculated free testosterone (pmol/L)	52 ± 25	51 ± 20	0.858	54 ± 25	54 ± 18	0.933
	(39 to 65)	(40 to 62)		(40 to 68)	(43 to 65)	
Dehydroepiandrosterone-sulphate (µmol/L)	7.3 ± 2.2	7.8 ± 3.9	0.636	7.1 ± 2.3	8.0 ± 4.0	0.463
	(6.2 to 8.5)	(5.7 to 9.9)		(5.7 to 8.4)	(5.5 to 10.6)	
Insulin sensitivity index	4.2 ± 2.2	4.2 ± 2.5	0.997	4.0 ± 1.9	4.2 ± 2.7	0.874
	(3.1 to 5.3)	(2.9 to 5.5)		(3.4 to 4.6)	(2.5 to 5.9)	

Continuous and discrete variables are shown as mean ± SD and counts (%), respectively. Figures below those statistics denote 95% confidence intervals (lower limit to upper limit). Continuous and dichotomous variables were compared by *t* and χ^2^ tests, respectively.

**Table 2:** Cardiovascular autonomic function tests values at baseline.

	All women	Control arm	Experimental arm	*P*
	(n = 33)	(n = 16)	(n = 17)	
E/I ratio	1.44 ± 0.45	1.41 ± 0.60	1.47 ± 0.26	0.837
	(1.28 to 1.60)	(1.09 to 1.73)	(1.34 to 1.60)	
Valsalva test	1.50 ± 0.28	1.49 ± 0.31	1.51 ± 0.26	0.850
	(1.40 to 1.60)	(1.33 to 1.66)	(1.38 to 1.64)	
30:15 ratio	1.62 ± 0.49	1.73 ± 0.65	1.51 ± 0.22	0.220
	(1.45 to 1.79)	(1.38 to 2.08)	(1.40 to 1.62)	
Resting heart rate (bpm)	72 ± 9	73 ± 9	72 ± 10	0.833
	(69 to 75)	(68 to 78)	(67 to 77)	
Change in systolic BP	3 ± 7	2 ± 8	3 ± 7	0.682
in response to standing (mmHg)	(1 to 6)	(− 2 to 6)	(− 1 to 7)	
Change in diastolic BP	4 ± 6	4 ± 6	5 ± 6	0.590
in response to standing (mmHg)	(2 to 6)	(1 to 7)	(1 to 9)	

Continuous and discrete variables are mean ± SD. Figures below those statistics denote 95% confidence intervals (lower limit to upper limit). Comparisons between both arms of the study were conducted by *t* tests.

**Table 3:** Differences between patients allocated to scheduled bloodletting compared to those allocated to the control arm in the frequencies of abnormalities in blood pressure regulation and indexes of cardioautonomic neuropathy.

Time (months)	Bloodletting arm			Control arm			Odds ratio^a^	95% confidence interval	*P*
	− 3	0	9	− 3	0	9			
*Office blood pressure*									
High-normal	0 (0%)	1 (6%)	1 (7%)	0 (0%)	1 (7%)	0 (0%)	0.958	(0.873–1.050)	0.354
Hypertension	1 (6%)	1 (6%)	0 (0%)	1 (6%)	0 (0%)	0 (0%)	nc	nc	nc
*Ambulatory blood pressure monitoring*									
Daytime hypertension	0 (0%)	0 (0%)	1 (10%)	1 (6%)	0 (0%)	1 (8%)	0.979	(0.828–1.158)	0.807
Nighttime hypertension	1 (6%)	0 (0%)	0 (0%)	0 (0%)	0 (0%)	1 (8%)	nc	nc	nc
24 h-hypertension	0 (0%)	0 (0%)	0 (0%)	0 (0%)	0 (0%)	1 (8%)	nc	nc	nc
Non-dipping pattern*^†^	4 (25%)	13 (93%)	4 (40%)	5 (31%)	11 (92%)	7 (58%)	1.012	(0.843–1.214)	0.901
*Cardioautonomic neuropathy*									
Mild	3 (18%)	2 (12%)	4 (29%)	2 (13%)	2 (13%)	1 (8%)	0.923	(0.763–1.116)	0.406
Definitive	0 (0%)	0 (0%)	0 (0%)	0 (0%)	0 (%)	0 (0%)	nc	nc	nc
Severe	0 (0%)	0 (0%)	0 (0%)	0 (0%)	0 (%)	1 (8%)	nc	nc	nc
Global	3 (18%)	2 (12%)	4 (29%)	2 (13%)	2 (13%)	2 (17%)	1.044	(0.860–1.267)	0.665

Data are shown as counts (%).

nc, Not computable.

*P < 0.001 for comparison between visits − 3 and 0 (run-in period) when considering all women as a whole; †*P* = 0.002 for comparison between visits 0 and 9 (experimental period) when considering all women as a whole.

^a^Control arm and month 0 visit were considered as reference categories in the binary logistic generalized estimating equations analyzing the experimental phase of the study.

### Changes observed during the run-in period when considering all patients as a whole

Twenty-six out of 33 women (78.8%) had valid ABPM recordings at the end of the run-in period (month 0). After 3 months of treatment with COC, we observed a statistically significant increase in office systolic BP values [MD 2.5 (0.3–4.8) mmHg] and night-time ambulatory systolic BP [MD 4.1 (1.4–6.8) mmHg] (Fig. 2). The percentage of women showing a non-dipping pattern in nocturnal BP was increased at the end of the run-in period with respect to baseline figures (28.1 vs 92.3%, *P* < 0.001) (Table 3). However, there were no significant changes in the percentage of women with a diagnosis of hypertension (Table 3).

**Figure 3:**
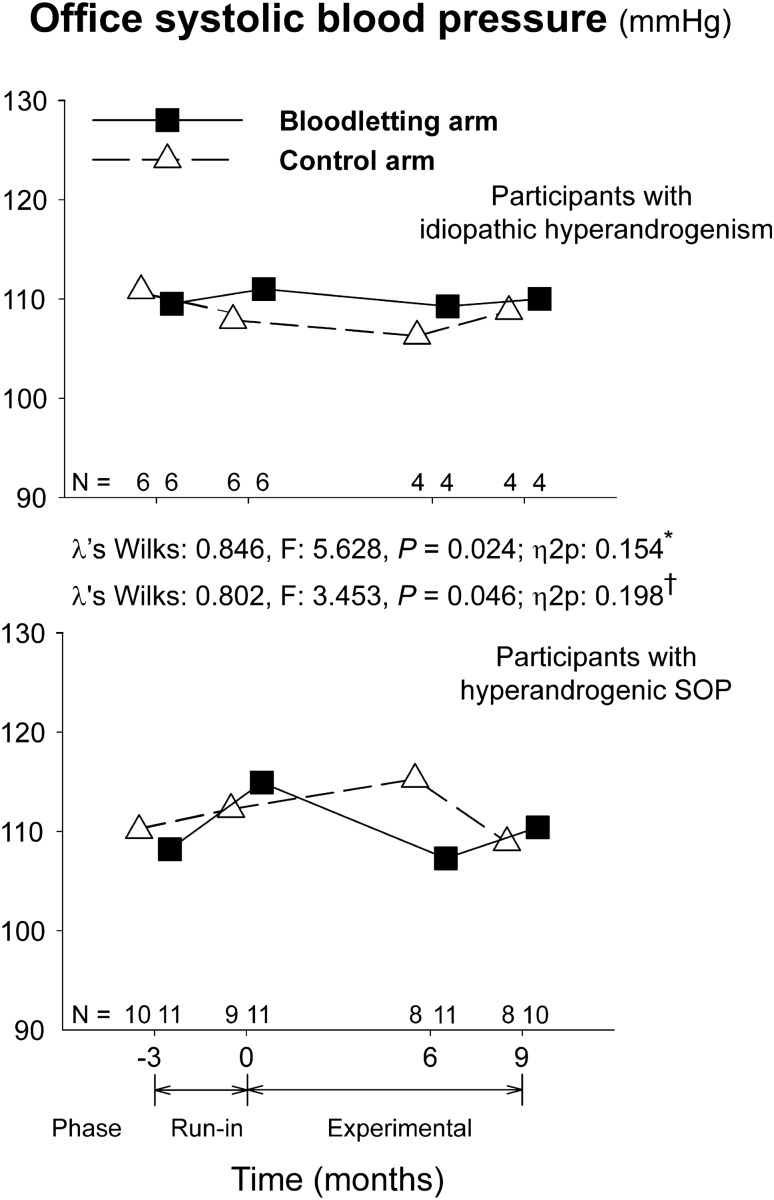
Influence of functional hyperandrogenism (hyperandrogenic PCOS vs idiopathic hyperandrogenism) on systolic blood pressure. Data are shown as means (SEM) of the patients remaining at each visit of the trial (figures above the x-axis) even though we conducted intention-to-treat statistical analyses. *Interaction with the visit of the study during the run-in period. †Interaction with the visit of the study and arm of treatment during the experimental phase of the trial.

After introducing the presence of obesity (defined by a BMI ≥ 30 kg/m^2^) as a between-subjects covariate, we also observed a significant increase in day-time ambulatory systolic BP [MD 2.7 (0.2–5.4) mmHg; λ’s Wilks: 0.876, F: 4.241, *P* = 0.048, η_p_^2^: 0.124]. However the interaction between obesity and the visit of the study did not reach statistical significance (λ’s Wilks: 0.991, F: 0.280, *P* = 0.600, η_p_^2^: 0.009) meaning that the influence of obesity was the same in both arms of the RCT. When introducing the type of hyperandrogenism (hyperandrogenic PCOS vs idiopathic hyperandrogenism) as a covariate, we found a significant interaction of the visit of the study with the changes observed in the office systolic BP during the run-in period (Fig. 3). Those women with hyperandrogenic PCOS suffered a significant increase in their values [MD 4.4 (1.7–7.2) mmHg], whereas office systolic BP did not change in their counterparts with idiopathic hyperandrogenism [MD − 0.8 (− 4.6 to 3.1) mmHg]. No other significant changes were observed in office BP, ABPM, hypertension, or CAN frequencies because of obesity or type of hyperandrogenism.

**Figure 4:**
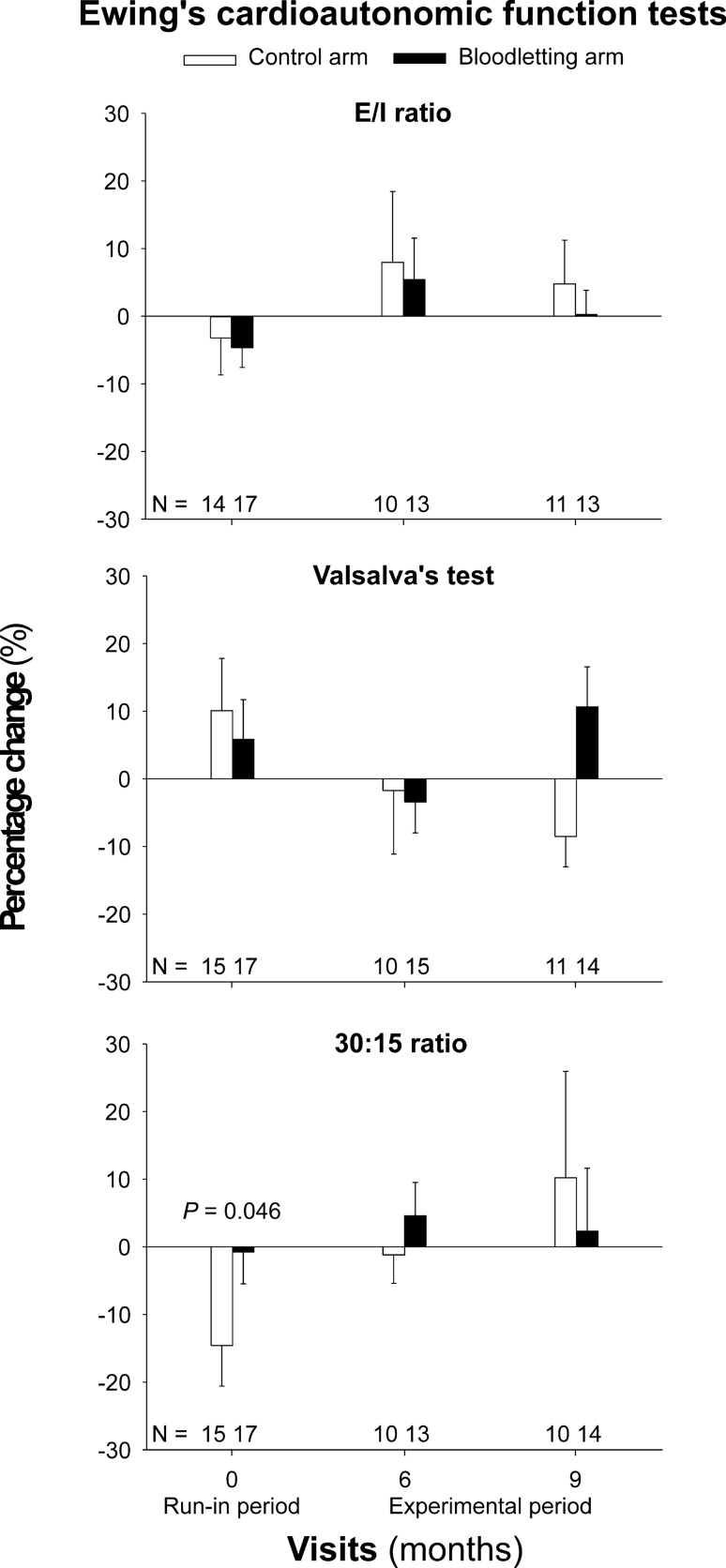
Percentage changes in the Ewing’s cardioautonomic function tests throughout the trial. Data are shown as means (SEM) of the patients remaining at each visit of the trial (figures above the x-axis) even though we conducted intention-to-treat analyses. Left side columns show the changes occurred during the run-in period (from months − 3 to 0). Central columns show the percentage change occurred from months 0 to 6 of the experimental period. Right side columns show the percentage change observed from months 0 to 9 of the experimental period.

During the run-in period, the 30:15 ratio significantly decreased with respect to baseline values [MD − 0.18 ± 0.51 (− 0.36 to − 0.00); Fig. 4, bottom panel]. At the end of the run-in period, no women had developed resting tachycardia or orthostatic hypotension.

### Primary outcomes

#### Blood pressure recordings

The mean time elapsed from phlebotomy procedures to BP assessments was 87 ± 59 days. Bloodletting was effective in decreasing body iron stores as previously reported^18^. Circulating ferritin levels decreased in the participants submitted to scheduled bloodlettings, whereas remaining unchanged in those allocated to observation (Table 4). During the experimental phase of the trial, there were no statistically significant changes in office systolic or diastolic BP values when all patients were considered as a whole (Fig. 2, top panel). We observed statistically significant interactions between study arm and visits of evaluation in both office systolic BP (λ’s Wilks: 0.784, F: 4.124, *P* = 0.026, η_p_^2^: 0.216) and office diastolic BP (λ’s Wilks: 0.645, F: 8.255, *P* = 0.001, η_p_^2^: 0.355). These interactions were caused by a slight decrease in systolic and diastolic BP values from month 0 to month 6 in the experimental arm followed by a mild increase from month 6 to the end of the study, whereas the women in the control arm showed opposite changes (Fig. 2, top panel). However, BP values at the end of the study were similar in both arms of treatment and almost identical to those observed at the beginning of the experimental phase of the study (Fig. 2, top panel). We did not observe any other statistically significant change throughout the trial in systolic or diastolic BP as measured by ABPM. Introducing the presence of obesity as inter-subjects covariate in the repeated-measures general linear model (GLM) did not change the lack of effect of bloodletting on BP recordings. Nevertheless, there was a significant interaction between having hyperandrogenic PCOS or idiopathic hyperandrogenism, the arm of treatment, and the visit on office systolic BP. This triple interaction consisted of a decrease in systolic BP values from month 0 to month 6 in the women with classic PCOS submitted to bloodletting followed by an increase from month 6 to the end of the study, whereas those women in the control arm showed opposite changes, and participants with idiopathic hyperandrogenism did not show any change throughout the trial independently of the arm of treatment (Fig. 3). We did not observe any other influence of the type of hyperandrogenism on the changes in BP recordings over the trial.

**Table 4:** Change in ferrokinetic parameters in each arm of the trial throughout the study.

Time (months)	− 3	0	6	9	Changes during run-in period	Effect of visit (experimental period)	Interaction of visit * study arm (experimental period)
					*P*	*P*	*P*
*Transferrin saturation* (%)							
Control group	26 ± 11	23 ± 8	20 ± 8	21 ± 12	0.223	0.209	0.210
Experimental group	27 ± 11	24 ± 11	23 ± 12	17 ± 7			
*Ferritin (µg/L)*							
Control group	67 ± 36	71 ± 39	77 ± 51	89 ± 93	0.005	0.067	0.003
Experimental group	68 ± 31	93 ± 49	39 ± 36	22 ± 11			
*Total iron binding capacity (µmol/L)*							
Control group	57 ± 8	51 ± 11	73 ± 14	75 ± 15	< 0.001	0.003	0.430
Experimental group	64 ± 8	71 ± 15	80 ± 15	85 ± 16			
*Iron (µmol/L)*							
Control group	14 ± 6	14 ± 5	12 ± 4	13 ± 6	0.864	0.913	0.393
Experimental group	15 ± 5	15 ± 6	15 ± 7	13 ± 3			

Continuous and discrete variables are mean ± SD.

Changes during the run-in period were analyzed by paired *t* tests considering all women as a whole. The effect of visit and the interaction between the visit and study arm of the study throughout the experimental phase of the trial were analyzed by repeated-measures general linear models.

#### Frequency of hypertension

There were no statistically significant changes in the frequency of office hypertension and of 24 h, nighttime, or daytime hypertension throughout the trial (Table 3). The percentage of patients with a non-dipping pattern significantly decreased during the experimental phase of the study when all women were considered as a whole [OR 0.694 (0.577–0.835), *P* < 0.001], although there were no differences depending on the study arm (Table 3). Such results remained unchanged after introducing the presence of obesity or type of hyperandrogenism as covariates in the generalized estimating equation (GEE) models.

### Exploratory outcomes

#### Cardiovascular autonomic function

There were no statistically significant changes in Ewing´s tests during the experimental period, either when all patients were considered as a whole or when considered as a function of the study arm (Fig. 4). We did not observe statistically significant changes in the frequency of CAN during the experimental period of the study either (Table 3). Only one woman who was allocated to the control arm presented with resting tachycardia at the end of the trial. Another woman allocated to the experimental arm presented with orthostatic hypotension at the month 6 visit, but showed a normal BP response to orthostatism at the end of the trial.

## Discussion

Phlebotomy has demonstrated to be useful for the sustained management of erythropoietin (EPO)-induced malignant hypertension in patients on chronic hemodialysis and post-transplant hypertension associated with erythrocytosis^19,20^. Insulin-resistant patients with metabolic syndrome and hypertension reduced office systolic and diastolic BP after repeated phlebotomies in parallel with a moderate reduction in body iron stores^13^. Furthermore, iron-mediated oxidative stress affects endothelium-dependent vasodilation^21^. Hence, a priori, an insulin-resistant condition such as functional hyperandrogenism that is associated with a mild iron overload and oxidative stress^9,22^, as well as with frequent BP abnormalities^2,4,23^, became a suitable setting in which to test the efficacy of iron depletion on BP outcomes.

Current COCs associate a very modest increase, if any, in the BP of most users from the general population. This appears to be also the case in women with functional hyperandrogenism^6,24^. However, the prevalence of hypertension in premenopausal women with PCOS is increased with respect to their non-hyperandrogenic counterparts^4,23^, even in the subset of patients with normal weight^25,26^. Despite being the mainstay of treatment in women with functional hyperandrogenism not seeking pregnancy, COCs are relatively contraindicated in women with preexisting hypertension, since COC users are at increased relative risk of myocardial infarction and stroke^27^. This risk is further increased when additional risk factors such as hypertension coexist^28^. The participants in our trial showed a slight increase in office and ambulatory systolic BP — with no changes in the frequency of hypertension — soon after beginning COC intake. This observation was specially marked in women with hyperandrogenic PCOS, and likely translates a more severe phenotype in them than in those participants with idiopathic hyperandrogenism. Besides, we found a dramatic increment in the percentage of women with a non-dipping pattern in their nocturnal BP recordings during the run-in period. This last finding is very relevant, since a blunted nighttime BP decline increases the risk of cardiovascular events, even in normotensive individuals^29^. The effect of COCs on sympathetic nerve activity is unclear in healthy women, although the nocturnal fall in BP, which is sympathetically mediated, might be affected by its use^30^. In conceptual agreement, the participants in our trial also showed a lower HR variability in response to standing after beginning COCs with respect to baseline values. Women allocated to scheduled blood donations did not obtain any particular effect of this intervention on their BP or cardioautonomic function tests. Notwithstanding, the use of a COC after the run-in period was accompanied by a significant reduction in the percentage of non-dippers at the end of the trial. In keeping, the percentage of women with a non-dipping pattern of BP did not show any significant change over 6 months of treatment with a COC containing EE plus CPA in a previous study from our group^6^. However, because we did not conduct ABPM at 12 weeks in that earlier trial, we may have missed fluctuations in the circadian BP rhythm at that specific point of follow-up as those here observed.

Abnormal nocturnal fall in BP relies on a harmed cardiovascular autonomic function^2^. We can speculate a short-term deleterious effect of a COC on our participants, supported by the above mentioned decrease in 30:15 ratios. However, the role of long-term COC use on dipping patterns clearly needs further research.

Participants with a more severe phenotype — i.e.: hyperandrogenic PCOS — showed a transient and mild benefit of iron depletion in their office systolic BP during the first 6 months of the trial; at the end of the study, however, we were not able to demonstrate any benefit of iron depletion on the BP in any study subgroup. Mechanisms of polyglobulia or EPO-induced hypertension include increased blood-viscosity and EPO-related vasoconstriction^19,20^. Chronic exogenous EPO administration also promotes vascular remodeling and hypertrophy maintaining BP elevation in the long-term^31^. While bloodletting may play a role in the management of these conditions by means of reductions in key hemorheological variables, blood viscosity may be a more subtle phenomenon in women with functional hyperandrogenism and/or those taking COCs^32^. In our study population, only 2 women allocated to the experimental arm had hyperfibrinogenemia (defined by plasma fibrinogen levels > 4.5 g/L) as a marker of hyperviscosity at baseline. After starting on COC, one of them normalized fibrinogen concentrations in the experimental arm, yet another 2 developed hyperfibrinogenemia in the control and experimental arms, respectively (data not shown). In the same way, no women presented with erythrocytosis (hemoglobin levels > 160 g/L and/or a hematocrit value > 0.48) at baseline, and only one women allocated to the control arm developed a mildly elevated hematocrit level (0.50) at the month 6 visit, that returned to normal values at the end of the study. Thus, hemorheological parameters neither appeared to be a major problem in our patients nor were modified by bloodletting.

In a previous RCT by others, bloodletting appeared to improve BP in patients with metabolic syndrome and hypertension^13^. Nonetheless, this trial has important differences with regards to our study population and research design. The men and women included in that trial^13^ were predominantly obese (mean BMI > 32 kg/m^2^), 62 out of 63 participants had a history of hypertension at study entry, and 38% of them presented with type 2 diabetes. Two phlebotomy sessions were performed in a month removing from 550 to 800 mL, and outcome measurements, including office BP, were obtained at week 6. Such a reduction in blood volume could induce hemodynamic changes leading to lower peripheral vascular resistances, thereby decreasing BP soon after the phlebotomy procedure. However, BP can increase again after 4–6 weeks as reported in patients with resistant hypertension treated by phlebotomy^33^. Since we re-evaluated our patients much later after phlebotomy was performed (87 days as a mean), we could have missed that transient drop in BP.

In the above mentioned trial, systolic BP improvement directly correlated with the decrease in ferritin levels after bloodletting^13^. Thus, the authors speculated on a beneficial effect of iron depletion on the vascular tone by reducing iron-mediated oxidative stress^13^. Those women submitted to blood donation in our RCT reduced successfully their circulating ferritin concentrations^18^ and, hence, an improvement in their redox homeostasis could be expected. However, such a hypothetical effect did not translate into BP changes in our women submitted to bloodletting.

Among the weaknesses of our study, we acknowledge: (1) even though our sample size was enough power to reveal significant changes in office systolic BP recordings, if they would exist, it might be likely inadequate to detect small differences in the frequency of blood pressure or cardioautonomic abnormalities. However, the actual figures found throughout the trial were virtually identical in both arms of treatment; (2) the same way, mild differences in some outcomes as a function of obesity subgroups might be overlooked because of our sample size; (3) we include patients with normal ferritin levels and without a prior diagnosis of hypertension, when those hypertensive women with hyperferritinemia could be more prone to obtain a benefit from scheduled phlebotomies; (4) as a consequence of blood donations or of restoring regular menses as many as 41% and 28% of women who underwent blood donations or observation, respectively, developed iron deficiency throughout the clinical trial^18^, and it is plausible that such a deficiency worsens redox homeostasis^34,35^; (5) we used a COC containing ethynil-estradiol (EE) plus cyproterone acetate (CPA) because of this combination is the only one currently approved for androgen excess symptoms by European and Spanish regulatory agencies. It is possible that another COC containing lower estrogenic doses and/or progestogenic compounds with different affinities for steroid receptors could show different impacts on BP; and (6) we studied a mixed population of women with functional hyperandrogenism and, therefore, possibility exists that the degree of severity in the androgenic phenotype influences the response to the interventions studied here, as suggested by our findings.

In summary, women with functional hyperandrogenism suffer from subtle abnormalities in BP regulation — such as a non-dipping pattern in the physiologic nocturnal decrease in BP and cardiovascular autonomic function as a consequence of the treatment with COCs. This is an issue of especial concern in these women in whom several cardiovascular risk factors cluster from early ages. Even though in theory a mild iron overload might play a pathophysiological role in these disturbances, in view of current evidence, scheduled phlebotomies cannot be recommended as an approach to overcome BP abnormalities in normoferritinemic women with PCOS or idiopathic hyperandrogenism taking COCs. Whether or not iron depletion by bloodletting may be beneficial for hyperandrogenic women with hyperferritinemia and/or hypertension at diagnosis, taking or not COCs, would need further research.

## Methods

### Study design

We conducted a parallel and controlled non-commercial RCT. The study protocol was registered at ClinicalTrials.gov (Identifier: NCT02460445. Date of first registration: 02/06/2015). The report of trial findings conforms to the Consolidated Standards of Reporting Trials (CONSORT) 2010 guideline^36^. The CONSORT guideline checklist is adhered to the supplementary data.

### Patients

Premenopausal women with functional hyperandrogenism^37^ — including hyperandrogenic PCOS (ie, clinical or biochemical hyperandrogenism plus ovulatory dysfunction and/or polycystic ovarian morphology), and idiopathic hyperandrogenism — aged 18–45 yr were consecutively recruited at the Reproductive Endocrinology clinic from an Academic Hospital in Madrid, Spain (Hospital Universitario Ramón y Cajal) (Fig. 1). The inclusion and exclusion criteria had been detailed elsewhere^18^. To be included in the study, none of the women had a prior history of dyslipidemia, hypertension, prediabetes, diabetes mellitus, gestational diabetes, or cardiovascular events, nor had been treated with COCs, antiandrogens, insulin sensitizers, or any drug that might interfere with BP regulation, lipid profile, or carbohydrate metabolism, or oral/parenteral iron therapy, for the previous 3 months.

### Randomization

We used stratified block randomization to allocate the patients (1:1) to scheduled bloodletting (experimental arm) or to observation (control arm). Blocks of 10 sealed opaque envelopes (5 per arm) served for treatment assignment. One investigator (M.L.-R.) generated the randomization envelopes, whereas another (A.E.O.-F.) enrolled and assigned the participants to their arm of treatment. No masking method was used after randomization.

### Intervention

The trial procedures have been described in detail elsewhere^18^. In short, study subjects completed a baseline assessment (month − 3 visit) after randomization that included BP measurements and tests of cardiovascular autonomic function. Then, they started a run-in period of treatment with a COC [21 pills containing 35 μg of EE plus 2 mg of CPA followed by 7 placebo pills, Diane^35^ Diario; Schering España S.A., Madrid, Spain]. After 3 cycles of treatment (month 0 visit) and a complete re-evaluation, we started the bloodletting intervention in the experimental arm consisting of 3 scheduled phlebotomies, drawing 500 mL (450 g) of blood in each of them (Fig. 1). Clinical, biochemical, BP, and cardiovascular autonomic assessments were again performed at month 6 and 9 visits (end of the study). Women in both arms of the clinical trial maintained their treatment with the COC until the end of the study.

### Outcomes assessment

Clinical, anthropometric, and biochemical evaluations have been previously reported^18^. At month − 3 visit, these evaluations were performed between day 3 and 9 of a spontaneous menstrual bleeding or after excluding pregnancy in amenorrheic patients. The same protocol was also repeated at month 0, 6, and 9 visits regardless of the day of the menstrual cycle because of all women were on the same COC. Two trained investigators (M.L.-R. and A.E.O.-F.) were responsible for the BP measurements and cardiovascular autonomic function tests, which were conducted after an overnight fast.

BP and heart rate (HR) were measured three times, 1–2 min apart, using a calibrated automatic digital sphygmomanometer (Welch Allyn Spot Vital Signs 4200B, Welch Allyn^®^, NY, USA) in the nondominant arm, with a proper cuff, and while the women were seated for at least 5 min before taking a reading. The average of the three measurements was used as an estimation of office systolic and diastolic BP, and HR at resting. For 24 h-ABPM, we used WatchBP 03 oscillometric devices (Microlife WatchBP AG, Widnau, Switzerland) with STRIDE BP approved validation, following the protocol previously described^2,4,6^. A minimum of 70% usable BP recordings were required for considering valid 24 h-ABPM measurements. Night-time was defined according to individual patients’ diary. The nocturnal decreases in systolic and diastolic BP were calculated using the equation [(mean of diurnal BP − mean of nocturnal BP)/mean of diurnal BP] × 100. Nondippers were defined as those subjects who did not show a reduction in mean systolic and diastolic BP by at least 10% from day to night, and the remaining subjects were considered as dippers. Office and 24 h-ABPM hypertension was defined according to the 2018 European Society of Hypertension guidelines^38^.

Cardiovascular autonomic function (parasympathetic innervation) was assessed by the tests proposed by Ewing and Clarke using a Monitor OneDx^®^ System (Qmed, Inc., Eatontown, NJ)^39,40^. In a room maintained at stable temperature, all study women rested in supine for 10 min between 7:00 and 9:00 AM. We measured HR variability during respiration (deep breath test) by calculating the ratio of the maximum and minimum HRs during six cycles of paced deep breathing Expiration/Inspiration (E/I) ratio. We assessed HR response to Valsalva’s maneuver (Valsalva test) by calculating the ratio of the longest R–R interval after the maneuver to the shortest interval during or shortly after the maneuver. The ratio of the longest R–R interval — found at approximately beat 30 — to the shortest interval — found at about beat 15 — after standing up (30:15 ratio) served for assessing HR responses to standing (orthostatism test). Adrenergic innervation was assessed by changes in BP and HR after 5 min of active standing with respect to the values recorded during resting while supine.

A fall of > 20 mmHg in systolic BP or > 10 mmHg in diastolic BP in response to standing defined orthostatic hypotension^41^. Resting tachycardia was defined by a HR > 100 beats per minute. The presence of cardioautonomic neuropathy was diagnosed by a modification of the Ewing’s score^39^, as previously reported^40^. A composite score ≥ 1 diagnosed CAN. CAN was also classified as early or mild with a Ewing’s score between 1 and 2, and as definitive when ≥ 2. Two or more abnormal HR variability tests and an abnormal BP test defined CAN as severe^40^.

### Biochemical and hormonal assays

The assays used for biochemical and hormonal phenotyping have been already reported^18^.

### Sample size

We used the online calculator provided by the Massachusetts General Hospital Biostatistics Center (http://hedwig.mgh.harvard.edu/sample_size/size.html) for power calculations. The inclusion of 33 women by ITT analysis would provide power (1 − β) above 85% at a two-side 0.05 significance level to detect a mean of the differences (MD) between arms of treatment of at least 16.5 mmHg in the change of office systolic BP. We estimated a common SD of 14.9 mmHg as previously reported after reducing iron tissue depots by means of phlebotomy in patients with the metabolic syndrome^13^.

### Statistical analysis

Data are shown as means (SD), MD, or n (%) as appropriate unless otherwise stated, with their respective 95% confidence intervals (CI) (lower limit to upper limit). For continuous variables, we assessed normality using the Kolmogorov–Smirnov test. We applied logarithmic transformation to ensure normality as needed. To assess the effect of COC administration during the run-in period, we applied paired *t* tests, despite mean changes in each arm are shown separately to rule out an unexpected behavior of some variables in any of them by visual inspection. The changes in the frequencies of hypertension or CAN during the run-in period were analyzed by McNemar’s tests.

To assess the effect of bloodletting on outcome variables, we implemented a repeated-measures GLM including the arm of treatment as between-subjects effect and the visit (month 0, 6, and 9) as the within-subjects effect. A statistically significant interaction of the between- and within-effects would indicate that the changes were different depending on the arm of treatment. We used Mauchly’s test to measure sphericity and applied Greenhouse–Geisser epsilon adjustment as needed. Repeated-measures GLMs were also used to address the influence of obesity and type of hyperandrogenism on the changes observed in BP introducing these variables as between-subjects covariates. The confidence intervals for MD in pairwise comparisons were adjusted for multiplicity by the Bonferroni method. The changes in the frequencies of hypertension or CAN during the study were analyzed by univariate binary logistic GEE models.

All inferential statistics were conducted by ITT. Participants who were randomized and completed the run-in period (from month − 3 to 0) comprised the ITT group, regardless of whether or not they completed the RCT. ITT analyses assumed that the dependent variables had not changed at the missing visits with respect to the values observed in the previous visit.

A nominal two-sided α level was set at 0.05. Statistical analyses were performed using PASW Statistics 18 (IBM España S.A., Madrid, Spain).

### Ethics approval and consent to participate

We obtained written informed consent from all participants in the study. The study protocol conformed to the ethical guidelines of the Declaration of Helsinki, and was approved by the Instituto Ramón y Cajal de Investigación Sanitaria ethics committee (Protocol ID#: 352/14; Date: November 28, 2014).

## Data Availability

The datasets used and/or analysed during the current study are available from the corresponding author on reasonable request.
